# Increased decision latency in alcohol use disorder reflects altered resting-state synchrony in the anterior salience network

**DOI:** 10.1038/s41598-021-99211-1

**Published:** 2021-10-01

**Authors:** Nicola Canessa, Gianpaolo Basso, Irene Carne, Paolo Poggi, Claudia Gianelli

**Affiliations:** 1 IUSS Cognitive Neuroscience (ICoN) Center, Scuola Universitaria Superiore IUSS, 27100 Pavia, Italy; 2 Cognitive Neuroscience Laboratory of Pavia Institute, Istituti Clinici Scientifici Maugeri IRCCS, Via Maugeri 4, 27100 Pavia, Italy; 3 University of Milano-Bicocca, 20126 Milan, Italy; 4 Medical Physics Unit of Pavia Institute, Istituti Clinici Scientifici Maugeri IRCCS, 27100 Pavia, Italy; 5 Radiology Unit of Pavia Institute, Istituti Clinici Scientifici Maugeri IRCCS, 27100 Pavia, Italy

**Keywords:** Cognitive control, Decision, Motivation, Addiction, Human behaviour

## Abstract

Increased decision latency in alcohol use disorder (AUD) has been generally explained in terms of psychomotor slowing. Recent results suggest that AUD patients’ slowed decision-making might rather reflect alterations in the neural circuitry underlying the engagement of controlled processing by salient stimuli. We addressed this hypothesis by testing a relationship between decision latency at the Cambridge Gambling Task (CGT) and intrinsic brain activity in 22 individuals with AUD and 19 matched controls. CGT deliberation time was related to two complementary facets of resting-state *f*MRI activity, i.e. coherence and intensity, representing early biomarkers of functional changes in the intrinsic brain architecture. For both metrics, we assessed a multiple regression (to test a relationship with deliberation time in the whole sample), and an interaction analysis (to test a significantly different relationship with decision latency across groups). AUD patients’ slowed deliberation time (p < 0.025) reflected distinct facets of altered intrinsic activity in the cingulate node of the anterior salience network previously associated with the “output” motor stage of response selection. Its heightened activity in AUD patients compared with controls, tracking choice latency (p < 0.025 corrected), might represent a compensation mechanism counterbalancing the concurrent decrease of its internal coherent activity (p < 0.025 corrected). These findings provide novel insights into the intrinsic neural mechanisms underlying increased decision latency in AUD, involving decreased temporal synchronicity in networks promoting executive control by behaviourally relevant stimuli. These results pave the way to further studies assessing more subtle facets of decision-making in AUD, and their possible changes with rehabilitative treatment.

## Introduction

Impaired decision-making might represent a core feature of addictions, promoting their development and mediating the adverse consequences of substance-related executive deficits on treatment adherence and relapses^1^. Addictions are considered to reflect the imbalance between bottom-up reward-related drives mediated by limbic structures such as amygdala and striatum, and altered executive control processes involving the anterior cingulate and prefrontal cortex^2^. This pattern might promote the onset of alcohol use disorder (AUD)^3^, and its detrimental effects on health and life expectancy^4^.

Within the framework of neuroeconomics, the development of addictions—including AUD—is indeed conceptualized in terms of maladaptive reinforced learning^5,6^, with alcohol use being associated to the rewarding experience of consumption and/or the omission of the aversive experience of craving^3^. In line with the evidence on AUD patients’ executive impairments^7,8^, the resulting shift from goal-directed to habitual behaviour is considered to be neurally mediated by the progressive dominance of hyperactive “reflexive” appetitive drives over hypo-active “reflective” mechanisms of executive control^2^.

An in-depth assessment of decision-making deficits and their neural bases is needed, however, to clarify the contribution of impaired choice to AUD. Most previous related attempts have used well-established tasks of decision-making under risk such as the Iowa Gambling Task^9,10^ and the Cambridge Gambling Task (CGT)^11^. The CGT requires participants to guess whether a token is hidden behind red or blue boxes presented in varying colour ratios (from 5-5 to 9-1), and then to place a bet (among a set of five pre-defined amounts) on the confidence in this judgment. The presence of multiple task-stages allows to disentangle different facets of decision-making, such as evaluation, adaptation of betting behaviour to outcome probability, and outcome feedback, reflecting in distinct patterns of brain activity. In particular, the dorsolateral prefrontal and anterior cingulate sectors of the executive network underpin the initial decision stage, while a key node of the reinforcement learning network such as the putamen^12^ is engaged in the subsequent betting stage^13^. At the behavioural level, the CGT provides distinct metrics of decision-making skills virtually unbiased by learning effects, while minimizing the loading on executive functions altered in AUD, such as working-memory^14^. Despite such control on potential confounding variables, previous studies comparing different types of addictions have suggested that increased CGT decision latency is specific to AUD^15,16^. While this evidence was generally explained in terms of AUD patients’ psychomotor slowing^17–19^, our recent data suggest that their longer deliberation time might reflect functional alterations in the neural circuitry underlying the engagement of controlled processes when behaviourally relevant, i.e. “salient”, stimuli are detected^20–24^.

One increasingly used approach to test such hypothesis is represented by resting-state functional MRI (RS-*f*MRI)^25^, highlighting intrinsic brain networks characterized by temporally coherent and spatially independent slow fluctuations of the BOLD signal even in the absence of sensory, motor or cognitive processing. The fast diffusion of this approach has been boosted by growing evidence of a spatial correspondence between such resting-state networks (RSNs) and the sets of brain areas underlying cognitive functions during task performance^26–28^. On this basis, distinct metrics of intrinsic brain functioning have been reported as neural markers of individual variability in cognitive, sensory or motor performance, both in normal^29,30^ and pathological^31,32^ conditions.

By coupling RS-*f*MRI with an extensive neuro-cognitive assessment, we have recently shown that AUD patients’ executive deficits reflect grey matter atrophy within, and altered intrinsic functional connectivity between, the fronto-insular and fronto-striatal brain networks underlying the salience-based transition from automatic to controlled processes^22,23^. We now aim to extend this evidence by assessing possible decision-making deficits in the same AUD sample, and their neural bases in the patterns of intrinsic brain functioning highlighted by resting-state *f*MRI. We thus related their CGT performance to complementary metrics of intrinsic activity which have been suggested as early biomarkers of functional changes in the intrinsic brain architecture^33^: spectral power of RSN timecourse and intensity of RSN spatial maps, reflecting the *coherence* and *strength of connectivity* of intrinsic intra-network activity, respectively.

Based on previous data from the same sample^24^, we predicted that AUD patients’ increased decision latency^15,16^ would reflect in altered metrics within the RSNs promoting the access to working-memory and executive resources^22,23,34^, and particularly in the anterior cingulate portion of the salience network driving the switch from default-mode to executive and motor networks^35,36^.

## Methods

### Participants

The overall sample included 22 adult AUD patients (9 females; mean age: 45.56 years ± 7.99; range: 29–58; mean education: 9.91 years ± 2.65) and 19 age- and education-matched healthy control subjects (8 females; mean age: 45.11 years ± 8.69; range: 27–57; mean education: 10.11 years ± 2.78). There was no significant demographic difference between patients and control subjects (Table 1), and a chi-square test confirmed that the distribution of males and females was not significantly different across groups (p = 0.9382). AUD patients’ inclusion criteria were: 1) AUD diagnosis based on DSM-5 criteria; 2) age between 20 and 60 years. For both groups, exclusion criteria were: major medical or neuro-psychiatric conditions, or comorbid disorders with the exception of nicotine dependence, prior loss of consciousness or brain injury, current use of psychotropic medications, contraindications to magnetic resonance imaging (MRI). Presence or history of alcohol abuse represented additional exclusion criteria for HCs. Patients were recruited during a 28-days inpatient alcohol withdrawal treatment, during which they underwent structured clinical interviews assessing their lifetime drinking history (type, amount and duration of alcohol consumption; days of abstinence before hospitalization; past use of other substances), blood chemistries and hematological tests alongside diagnostic testing, dietary visit, and motivational enhancement group therapy. At study enrollment they had been detoxified for at least 10 days, and the MRI session was performed at least 8 days after the end of benzodiazepine treatments. We screened controls through a cut-off of average alcohol intake < 1 standard unit of alcohol (UA) for females and 2 UA for males (one UA: 12 g of ethanol, e.g. 40 ml hard liquor, 125 ml wine or 330 ml beer) (Table 1). HCs were requested to remain abstinent since 10 days prior to MRI, and interviewed before the scanning session to assess compliance with this requirement.

**Table 1:** Demographics and alcohol use variables.

Demographic variables (HC and AUD)	Age (years)	Education (years)	Smoking status (yes/no)	
HC: Mean (SD)	45.11 (8.69)	10.11 (2.78)	6/13	
AUD: Mean (SD)	45.56 (7.99)	9.91 (2.65)	18/4	
p-value	0.426	0.405	< 0.01	

Alcohol use variables (AUD only)	Duration of alcohol use (years)	Average daily alcohol dose (UA)	Abstinence before MRI (days)	Past use of other substances
Females: Mean (SD)	11.89 (7.11)	14.94 (5.92)	14.22 (5.04)	None
Males: Mean (SD)	10.11(7.48)	14.18 (7.12)	18.92 (17.49)	Marijuana (n = 1); cocaine and marijuana (n = 2)
p-value	0.576	0.791	0.44	

The top table section reports the mean and standard deviation (SD) of demographic variables and smoking status for healthy controls (HC) and patients with alcohol use disorder (AUD), alongside the results of group comparisons with two-sample t-tests and a chi-square test. In the bottom part, duration of alcohol use, average daily alcohol intake and days of abstinence before MRI are reported separately for male and female AUD patients, alongside the results of gender comparisons with two-sample t-tests. Alcohol consumption was calculated as the average number of daily standard units of alcohol (UA) (one UA: 330 ml beer, 125 ml wine, or 40 ml hard liquor, corresponding to 12 g of ethanol).

*DF* degrees of freedom, *UA* units of alcohol.

All participants gave their informed consent to the study, that was approved by the ethics committee of ICS Maugeri scientific institute (Pavia, Italy) and carried out according to the relevant guidelines and regulations (Declaration of Helsinki).

### Decision-making assessment

We used the CGT^11^ to assess decision-making skills in AUD patients and HCs. This task provides distinct metrics of risk-taking behaviour outside a learning context, because all the relevant information, including the probability of potential gains and losses, is explicitly presented^14^. In each trial 10 boxes are shown, either red or blue with different ratios (from 5:5 to 9:1). Subjects are asked to select within 2 s the colour which is more likely to hide a token, and then to place a bet on the confidence in this judgment among a set of five pre-defined amounts (5%, 25%, 50%, 75% and 95% of their current total score). The amount bet is selected by pressing a button during either an ascending or descending series of values, and will be then added to/subtracted from the current score in case of correct/incorrect identification of the yellow token.

The CGT provides 6 outcome variables, reflecting distinct aspects of decision-making: (1) time spent for making a selection (deliberation time); (2) proportion of trials in which the participant selects the correct colour outcome (quality of decision-making; QDM); (3) difference in percentage bet in ascending vs. descending trials (delay aversion; DA); (4) mean proportion of points bet across all trials (overall proportion bet; OPB); (5) extent to which betting behaviour is moderated by the ratio of boxes (risk adjustment; RA); (6) proportion of points bet on trials in which the most likely outcome was chosen (risk taking; RT). Each of these measures is reported either for the single levels of the “order of presentation” (ascending or descending) and “ratio of the boxes” (5:5, 6:4, 7:3, 8:2, 9:1) factors, or as their grand average. Our analyses are based on the latter value, which is most representative of the overall subject’s performance.

### Analysis of CGT data

After assessing the normality of their distribution in the whole sample, we used Spearman’s correlation index and Mann–Whitney t-test to examine, respectively, age and group effects on each of the six CGT measures. We additionally performed exploratory analyses to test a group-by-sex interaction on CGT performance. For the CGT metric(s) showing a significant effect, we assessed a relationship with duration of alcohol use, amount of alcohol intake or abstinence duration in patients. Statistics were thresholded at p < 0.05 (one-tailed due to previous reports of altered CGT performance in AUD^15,16^).

### Resting-state *f*MRI study

#### RS-*f*MRI: data acquisition and pre-processing

We used a 3 Tesla General Electrics Discovery scanner (GE Healthcare) and a 16-channels head coil to collect anatomical (3D T1-weighted IR-prepared FSPGR; 152 slices, resolution = 0.9375 × 0.9375, thickness = 1 mm) and functional (240 echo-planar-imaging (EPI) volumes; TR = 2000 ms, resolution = 3 × 3 mm, thickness = 4 mm, inter-slice gap = 0.2 mm) MRI images. Participants were instructed to lie still, with their eyes open fixating a white cross on black background^37,38^.

Through a standard pre-processing of *f*MRI volumes, performed with SPM12 (http://www.fil.ion.ucl.ac.uk/spm), data were corrected for slice-timing, spatially realigned to the first volume and unwarped, spatially normalized to the Montreal Neurological Institute (MNI) space^39^ and resampled in 2 × 2 × 2 mm^3^ voxels, and spatially smoothed using a 8 mm gaussian isotropic kernel.

We used different approaches to address the effect of head motion on resting-state *f*MRI data^40,41^. First, the 6 SPM realignment parameters (3 translations + 3 rotations) were used to compute a comprehensive index of scan-to-scan head motion (i.e. framewise displacement) via the Motion Fingerprint toolbox^42^. Only for two patients this procedure highlighted few isolated volumes (1 and 3, respectively) exceeding a pre-defined threshold of 2 mm, which were removed by interpolation. While no significant group difference was found (controls’ mean: 0.107 mm ± 0.045; patients’ mean: 0.116 mm ± 0.058; t(38) = 0.57, p = 0.569), scan-to-scan head motion was modelled as nuisance regressor in statistical analyses to remove from results its residual effect (see “RS-fMRI statistical analyses”).

We then performed a spatial group independent component analysis (gICA), using the Infomax algorithm as implemented in GIFT (https://trendscenter.org/software/gift/)^43,44^, to extract 75 maximally independent and temporally coherent spatial sources, i.e. functional networks or “spatial maps”, from resting-state timecourses of the whole sample. Reliable RSNs were distinguished from physiological artifacts via visual inspection (to reject components with peak activations clearly involving tissues and structures other than grey matter), spectral characteristics of IC timecourses (to retain components dominated by low frequency fluctuations), and an Iq index reflecting the consistency of component extraction through 250 gICA rounds. The resulting 57 resting-state components^23^ were anatomically labelled based on a template including the main RSNs^26,43^ (Supplementary Tables S1–S4).

#### RS-*f*MRI statistical analyses

Statistical analyses were aimed to unveil the relationship between the CGT measure(s) showing a significant difference across AUD patients and controls and two complementary resting-state metrics^43^: (1) spectral power of RSN timecourse, i.e. the degree to which each frequency bin contributed to the spontaneous fluctuations of the BOLD signal, which reflects the *coherence of intra-network activity* (maximal with highest power at low frequencies); (2) the intensity of RSN spatial maps, reflecting the *degree of intra-network coactivation* and *connectivity*. Based on behavioural results (see “Decision-making performance”), we modelled only CGT deliberation time, which previous studies had already shown to be significantly higher in AUD patients than controls^15,16^.

For both resting-state metrics we modelled: a two-sample t-test (to asses group differences), a multiple regression (to test a relationship with CGT deliberation time in the whole sample), and an interaction analysis (to test a significantly different relationship with decision latency between groups). To this purpose, we modelled in GIFT univariate tests including group, decision latency and their interaction as covariates of interest, alongside nuisance predictors coding age, sex, smoking status, average head motion and total intracranial volume to remove their possible effect from results. Partial correlation coefficients tracked the relationship between each metric and each predictor of interest after removing the effect of nuisance variables. This procedure highlighted the frequency bins (or voxels) in which spectral power (or the intensity of activation) was associated with the effect of group, CGT deliberation time or their interaction. We additionally performed exploratory analyses to assess a possible modulation of these effects by sex. Namely, we assessed a two-way interaction between sex and group, and a three-way interaction between sex, group and decision-latency, on the aforementioned resting-state metrics.

Although behavioural analyses had shown no significant association between CGT deliberation time and duration of alcohol use, abstinence duration or amount of alcohol intake in patients (see “Decision-making performance”), we performed ancillary control analyses to assess their possible relationship with neuroimaging findings. To this purpose, we first extracted the individual parameters reflecting the spectral power (or intensity of activation) of the frequency bins (or voxels) showing statistical significance in at least one of the above effects of interest. We entered the resulting values in offline analyses aimed to assess their relationship with duration or amount of alcohol use, and abstinence duration.

Since two distinct statistical models were assessed, we set the statistical threshold at p < 0.025, with False Discovery Rate (FDR^45^) correction for multiple comparisons. In the GIFT framework, FDR corrects for the number of bins in power spectra and voxels in spatial maps.

## Results

### Decision-making performance

Compared with controls, AUD patients displayed a significantly longer deliberation time (U = 130, p = 0.019) (Table 2), which was not correlated with age, duration or amount of alcohol intake, nor with abstinence duration. No other CGT metrics displayed a significant group difference, and no metric was associated with a significant group-by-sex interaction.

**Table 2:** CGT performance.

CGT variable	HC mean (SD)	AUD mean (SD)	U	p-value
CGT-Quality of decision-making	0.847 (0.199)	0.834 (0.186)	187.5	0.289
CGT-Delay aversion	0.312 (0.389)	0.291 (0.252)	181.0	0.238
CGT-Overall proportion bet	0.512 (0.139)	0.488 (0.132)	189.0	0.307
CGT-Risk adjustment	0.900 (1.251)	0.628 (0.973)	185.0	0.271
CGT-Risk taking	0.536 (0.144)	0.509 (0.15)	193.0	0.344
CGT-Deliberation time (ms)	1968.385 (746.113)	2437.073 (837.1)	130.0	0.019

For each of the 6 CGT metrics, the table reports the mean outcome (with its standard deviation) in healthy controls and individuals with AUD, alongside the results of group comparisons with a non-parametric Mann–Whitney U-test.

### Neuroimaging results

#### Resting-state networks

A review of gICA results based on spectral profiles and visual inspections led to retain 57 components as RSNs. As previously reported^23^, we identified all the main known resting-state networks, i.e. default-mode, dorsal attentional, anterior and posterior salience, executive control, temporal-language, visual, sensorimotor, auditory, limbic, basal ganglia and cerebellum networks (Supplementary Tables S1–S4 online).

#### RS-*f*MRI results: coherence of activity

**Figure 1:**
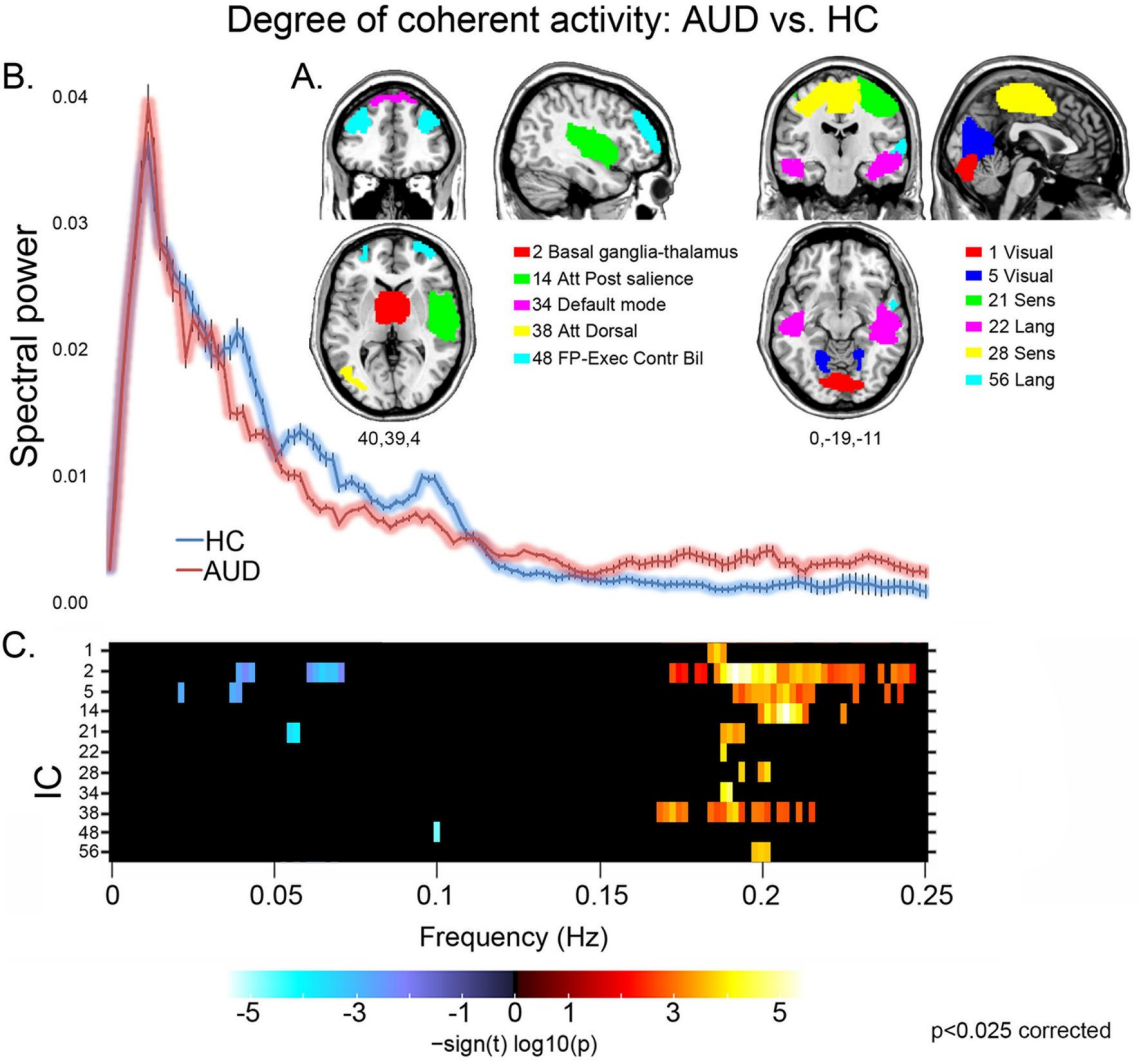
Coherence of intra-network activity: group comparison. For the Independent Components (ICs) showing a significant group effect (**A**), its direction and strength are depicted by the frequency bins reported in the bottom panel (**C**) (p < 0.025 corrected). A decrease of intra-network coherent activity in AUD patients compared with controls is depicted by a reduction of low frequency spectral power (< 0.1 Hz; blue colour scale), increase of high frequency spectral power (> 0.1 Hz; red colour scale), or both. In the middle panel (**B**), the line plots of spectral power depict the grand average, across all the significant ICs reported in panels A and C, of mean (± standard error) spectral power along the whole frequency band for AUD patients (pink) and controls (light blue). *Att Dorsal* dorsal attentional network, *Att Post salience* posterior salience network, *FP-Exec-contr* Fronto-parietal executive control network.

We observed distinct facets of faster BOLD fluctuations in AUD patients, indicating *lower coherent activity* than controls^43^, in several networks (Fig. 1). Namely, patients displayed both reduced spectral power at low (≤ 0.1 Hz) and very low (< 0.05 Hz) frequencies, and enhanced power at medium-to-high frequencies (> 0.1 Hz), in visual (1,5), sensorimotor (21), bilateral executive (48) and basal ganglia (2) networks. In other components we observed only increased high frequency power, in patients compared with controls, in the default mode (34), dorsal attentional (38), sensorimotor (28) and temporal-language (22,56) networks, alongside the posterior sector of the salience network (14). Importantly, findings concerning high-frequency spectral power should be interpreted cautiously, because of their possible artefactual nature associated with motion or physiological (e.g. respiratory) noise. On the other hand, the exclusion of noise components, the inclusion of head-motion as nuisance variable in statistical models, and the observation of high-frequency spectral power only in a subset of the modelled components (often showing also a concurrent decrease of low-frequency spectral power), suggest that—rather than physiological artifacts—this evidence might represent a facet of decreased coherent activity in AUD.

**Figure 2:**
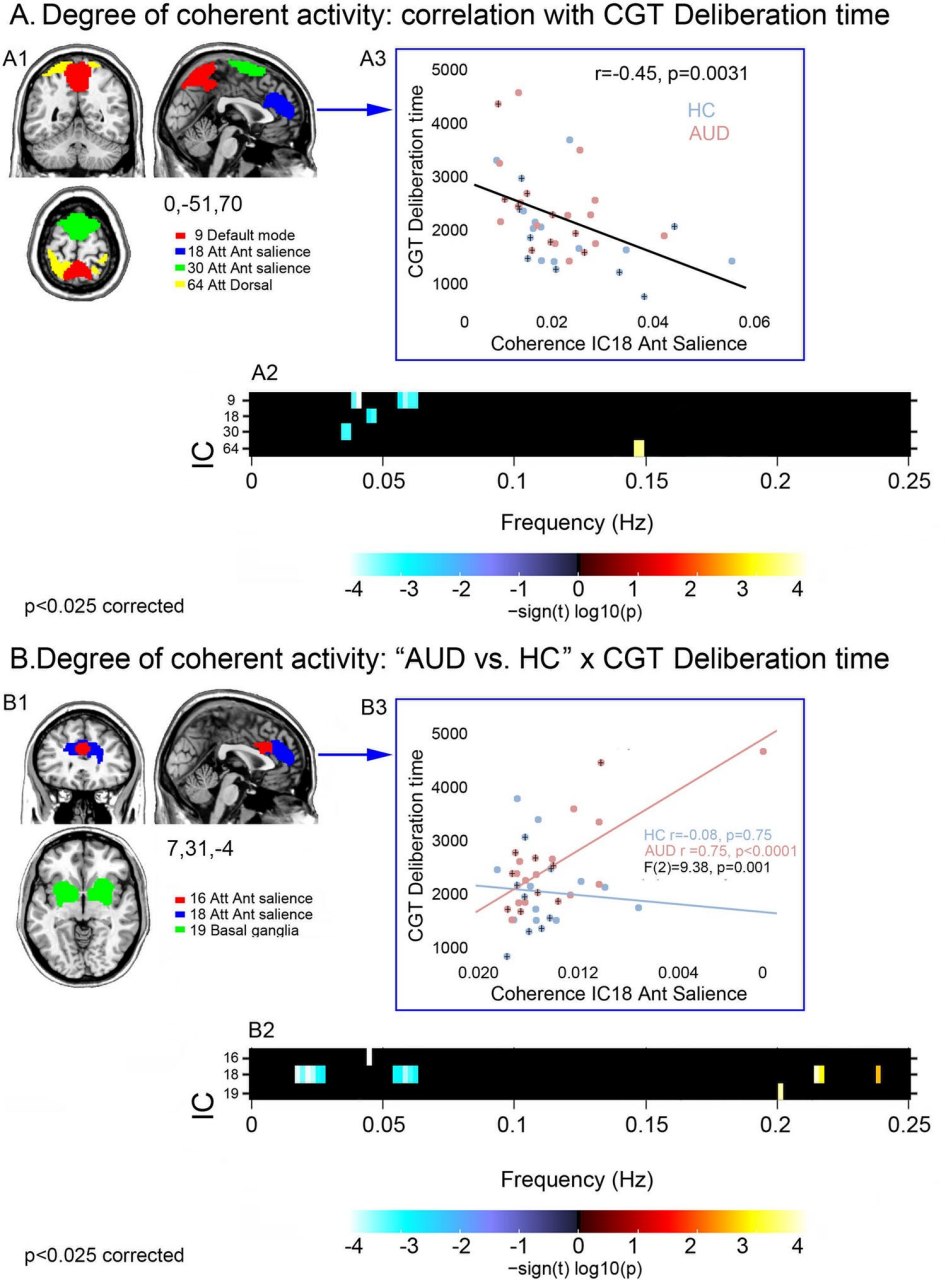
Coherence of intra-network activity: correlation with CGT decision latency in the whole sample and latency-by-group interaction. For the ICs showing a significant correlation with deliberation time (panel **A1**), or a latency-by-group interaction (panel **B1**), their direction and strength are depicted by the frequency bins reported in the respective panels A2 and B2 (p < 0.025 corrected). In the whole sample (**A2**), the association between decision latency and coherence of intra-network activity involves either a negative correlation with high coherent activity (low frequency power; blue colour scale) (9,18,30), or a positive correlation with low coherent activity (high frequency power; red colour scale) (64). Latency-by-group interaction analyses (**B2**) showed that the relationship with decision latency is stronger, in AUD patients vs. controls, in the same anterior salience network (16,18), as well as in the basal ganglia (19) networks. Scatterplots depict the relationship between decision latency and coherent activity in the anterior salience network, regardless of group (correlation analysis; **A3**) and specifically in AUD vs. controls (interaction analysis; **B3**). In the scatterplots, a “plus” (+) sign denotes female participants. Att Ant salience: anterior salience network; Att Dorsal: dorsal attentional network.

In the whole sample, decision latency was negatively related to the degree of coherent activity in the dACC portion of the anterior salience network (18,30), as well as in the posterior sector of the default-mode (9) and dorsal attentional (64) RSNs (Fig. 2A and Supplementary Fig. S1). In these networks, the relationship with longer decision latency reflected either decreased low-frequency power (9, 18,30) or increased high-frequency power (64).

A significant latency-by-group interaction highlighted several networks in which the degree of coherent activity displayed a steeper negative relationship with deliberation time in AUD patients than controls (Fig. 2B and Supplementary Fig. S2). In the former group, more than in controls, slower decision-making reflected reduced coherent intrinsic activity in the dACC sector of the anterior salience network (16,18). An opposite pattern was observed in the basal ganglia network (19), but with non-significant correlations in the two groups separately. This stronger relationship with AUD patients’ decision latency involved distinct aspects of decreased coherent activity, i.e. either reduced low-frequency power (16), enhanced high-frequency power (19), or both (18) (Fig. 2B). Importantly, when the same components were involved (i.e., 18), interaction analyses highlighted frequency bins close to, but other than, those highlighted by the respective correlation analyses. Moreover, this interaction remained significant even when excluding one potential outlier (p-corrected = 0.025).

#### RS-*f*MRI results: intensity of activity

**Figure 3:**
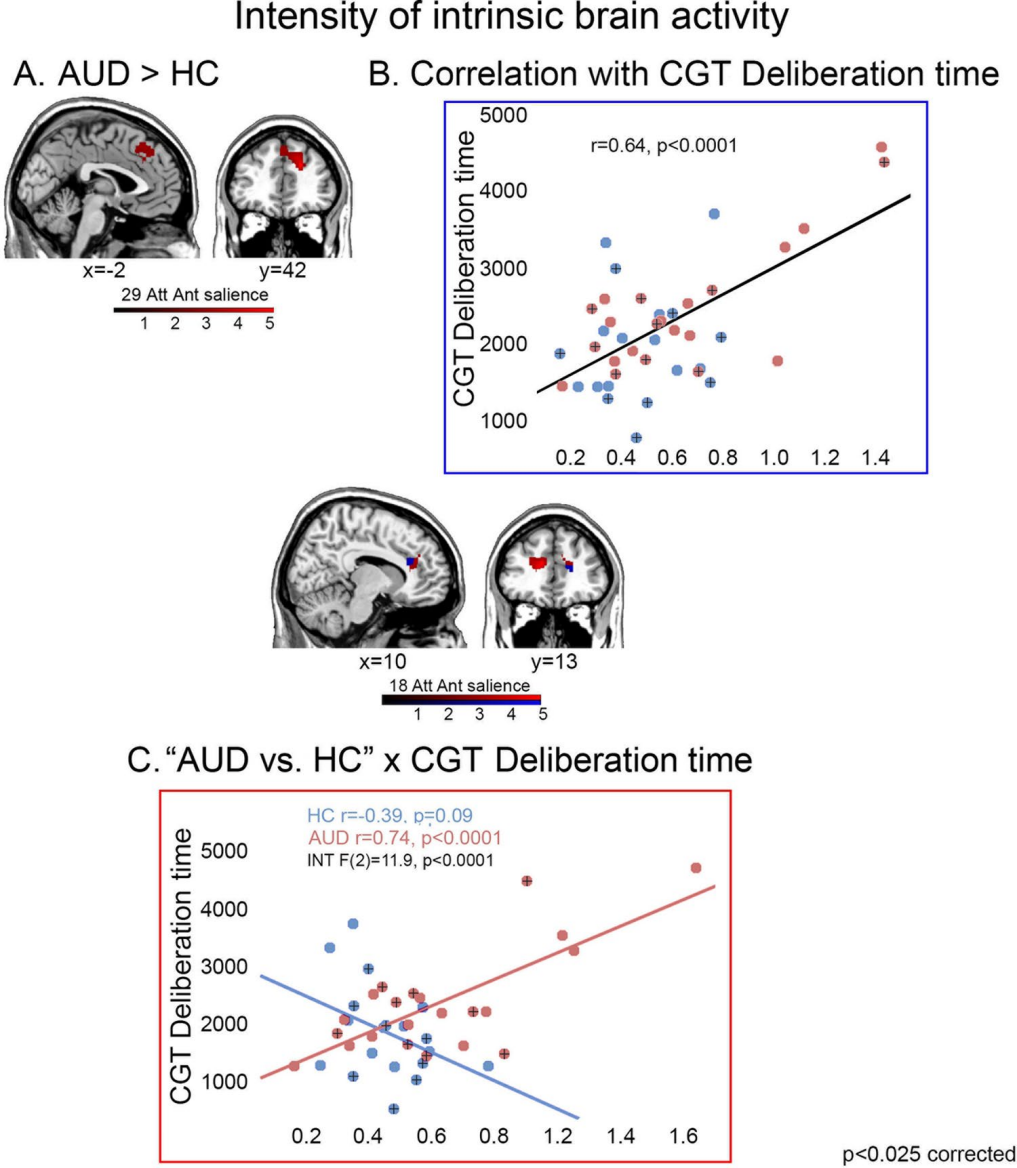
Intensity of intrinsic brain activity: group comparison and latency-by-group interaction. The figure depicts the resting-state components in which intrinsic activity was significantly stronger in AUD patients than controls (29; panel **A**), those showing a significant correlation between intensity of activation and decision latency regardless of group (blue color in component 18; panel **B**), and those displaying a significantly different relationship between intensity of activation and decision latency in AUD patients vs. controls (red color in component 18; panel **C**) (p < 0.025 corrected). Colour bars denote the direction and strength of these effects. In the scatterplots, a “plus” +) sign denotes female participants. *Att Ant salience* anterior salience network; *INT* interaction.

We found evidence of altered intensity of intrinsic activity*,* reflecting *intra-network connectivity and degree of coactivation*^43^, in AUD. As previously reported^23^, patients displayed stronger intrinsic activity than controls in the dACC sector of the anterior salience network (29) (Fig. 3A). In the whole sample, deliberation time was positively related to the strength of intrinsic activity in the dACC sector of another component encompassing the anterior salience network (18) (Fig. 3B). An adjacent, but distinct, sector of the same dACC component 18 displayed a significant latency-by-group interaction, i.e. a stronger association between increased deliberation time and strength of activity in patients than controls (Fig. 3C). Again, in the case of component 18 the latter analysis highlighted frequency voxels close to, but other than, those resulting from the respective correlation analysis. This interaction remained significant even when excluding one potential outlier (p-corrected = 0.004).

#### Sex effect and correlation with alcohol use variables

There was no significant relationship between duration of alcohol use, abstinence duration or amount of alcohol intake and either intra-network coherent activity, or intensity of activation, in the aforementioned components. Moreover, neither a two-way interaction between sex and group, nor a three-way interaction between sex, group and decision-latency, resulted in significant effects on the aforementioned resting-state metrics.

## Discussion

We coupled the CGT with resting-state *f*MRI to investigate decision-making performance, and its association with different metrics of intrinsic brain activity/connectivity, in early-abstinent AUD patients compared with age- and education-matched healthy controls. By minimizing demands for executive functions known to be impaired in AUD^14^, the CGT is expected to alleviate the possible confounding effects of the executive impairment which, in this sample, we have previously related to altered intrinsic activity in the dorsolateral prefrontal and striatal nodes of the executive control network^23^.

As predicted, AUD patients displayed longer deliberation time than controls^24^. While previous related evidence^15,16,46^ was explained in terms of AUD patients’ psychomotor slowing^17–19^, here we addressed the hypothesis that their increased decision latency might reflect functional alterations in the networks underlying the salience-based switch from automatic to controlled processing^22,23,34^.

This hypothesis was first supported by the present evidence on spectral power of RSN timecourse and intensity of RSN spatial maps, reflecting the level of intrinsic *intra-network* coherent activity and connectivity, respectively. Compared with controls, AUD patients displayed faster BOLD fluctuations within components encompassing default mode, attentional, salience, executive and striatal brain networks (Fig. 1). Based on the assumption that low-frequency fluctuations support temporal synchronicity among functionally related regions, a decrease of intra-network functional coherence is considered to reflect inter-network communication changes^31,47^, in turn resulting in altered cortical activity and cognitive functioning. In line with this notion, faster fluctuations have been described in neurological^32^ and psychiatric^31,47,48^ diseases, in which condition-specific impairments were suggested to reflect an altered interplay between functionally-related networks. In keeping with our previous morphometric evidence^22^, these data support the notion of a global brain damage in AUD, with cognitive impairments resulting from widespread alcohol-related structural and functional changes^49^. The stronger activity displayed by AUD patients in the prefrontal portions of the salience network (Fig. 3A) might thus reflect compensatory mechanisms, counteracting the decreased efficiency of the neural circuitry underlying cognitive performance^23,50^.

We addressed this hypothesis by focusing on decision-making performance, and particularly on the patterns of altered intrinsic brain functioning tracking AUD patients’ increased decision latency. To this aim, we tested both quantitative and qualitative differences across groups in the relationship between CGT decision latency and the aforementioned metrics of intrinsic brain activity, via correlation and latency-by-group interaction analyses, respectively.

Regardless of group, longer deliberation times reflected in decreased coherence of intra-network activity in several networks including the dACC sector of the anterior salience network, the posterior cingulate sector of the default-mode network and the dorsal attentional network. As shown by the representative scatterplot in Fig. 2A, the lack of group differences in the slope of this relationship highlights quantitative differences across AUD patients and controls along a continuum from normal to pathological conditions. The same trend was found for the intensity of intrinsic activity, related to the degree of *intra-network connectivity*: again, this relationship involved the anterior salience network, in which slower decision-making reflected in stronger intrinsic activity in the whole sample (Fig. 3B). The combination of these findings appears to highlight distinct but related facets of the neural mechanisms underlying increased decision latency in AUD.

First, longer decision latency reflected in decreased coherent activity within networks driving the transition—prompted by the detection of behaviourally salient stimuli—from default-mode to attentional and executive processes^51^. This finding fits with our previous evidence that an executive deficit in AUD, mostly involving visuomotor speed and attention, relates to decreased grey matter atrophy within, and functional connectivity between, the fronto-insular and fronto-striatal networks promoting the transition from automatic to controlled processing^22,23^. While our previous studies have shown the role of the fronto-insular cortex, however, here decision latency was specifically associated with the dACC portion of the anterior salience network. This differentiation fits with the notion that, in contrast to the anterior insula, the dACC salience node is more tightly involved in conflict monitoring and action selection via a rapid access to the motor system^34–36,52^. This circuitry is thus well suited to modulate, in addition to attention, also motor responses to salient stimuli such as gambles, which might explain the selective relationship between speed of decision-making and degree of intrinsic coherent activity in dACC.

Indeed, longer deliberation time was also reflected in stronger intensity of intrinsic activity in the same dACC component of the anterior salience network. Overall, this evidence fits with the notion that altered mechanisms of *salience attribution*, in addition to executive control, represent core features of addiction^53^. This view has been supported by task-*f*MRI studies, showing an enhanced response of the salience network in stimulant users^54^, and by preliminary evidence on resting-state activity, suggesting a disengagement of the salience and executive networks during abstinence compared with substance administration^55^. While fitting with our previous data on decreased fronto-striatal connectivity in the same sample^23^, the latter finding suggests that AUD patients’ overactivation in the anterior salience network might represent a neural compensation mechanism, counterbalancing the concurrent decrease of its internal coherent activity. Given the key role of the salience network in redirecting attentional resources and promoting action selection^35,52^, decreased coherent activity and increased strength of connectivity within its dACC sector seem thus to represent related facets of the neural bases of AUD patients’ slowed decision-making.

Importantly, none of the components in which resting-state metrics tracked deliberation time were significantly altered in the AUD group. This finding might be suggestive of exclusively quantitative, rather than qualitative, differences across groups in the relationship between spontaneous brain activity and decision latency. Notwithstanding their significant structural damage in terms of grey matter atrophy^22,24^, intrinsic activity in the default-mode, salience and executive networks would thus support action selection in decision-making, proportionally to the degree of their functional preservation, along a continuum involving both patients and healthy individuals. However, interaction analyses additionally highlighted *qualitative* group differences in this relationship, involving components specifically related to decision latency in AUD patients compared with controls. Once again, a common finding to these analyses was represented by the engagement of the dACC sector of the anterior salience network, in which both the degree of coherent activity and the intensity of activation displayed a steeper relationship with decision latency in AUD patients compared with controls (Figs. 2B and 3C). In line with the direction of the aforementioned effects, patients displayed a stronger association than controls between decision latency and both reduced coherent activity and increased strength of activation in the anterior salience network.

Overall, the present findings from an early-abstinent sample fit with growing evidence of neuro-cognitive and structural alterations in treatment-entering AUD patients^56,57^, which appear to undergo a considerable reversal with long-term abstinence^58–63^. Instead, the degree of fronto-striatal altered activity in AUD patients entering inpatient treatment has been shown to reflect the number of days of abstinence, and to predict heavy drinking behaviour during the subsequent outpatient treatment^64,65^. The lack of a significant relationship between CGT performance and abstinence duration in our AUD sample suggests that more sensitive metrics of decision-making and/or executive skills might be needed to detect and monitor the progression of recovery after abstinence onset. Moreover, the available literature highlights the importance of longitudinal measurements to assess the extent to which such metrics are predictive of relapse after treatment^64^.

These considerations highlight some limitations of this study. Although its previous administration in different populations makes the CGT a well-established reference for assessing the quality of choice^13,66^, this task provides a relatively rough measure of the skills subsumed under the umbrella notion of “decision-making under risk”. While this study was aimed to unveil the neural bases of the previously reported increased decision latency in AUD patients^15,16^, future extensions are thus expected to take advantage of the present evidence as a basis to address other possible, and more subtle, facets of altered decision-making in AUD. In addition, the inclusion of smoking status as covariate in statistical analyses does not allow to exclude its possible residual contribution to some of the reported differences across patients and controls. Moreover, the ICA approach entails the extraction of resting-state components from both healthy controls and patients, to ensure that the same components can be compared across groups. While simulation-based studies have shown the capability of this approach to capture between-subject differences (44), its drawback is the additional noise carried by patients’ data. It should thus be considered that, in the present study, at least a portion of the significant components were likely mixtures of signal and noise. Finally, the present findings are limited by a small-to-moderate sample size. While this limitation is partially mitigated by a careful control of potential confounding variables, via age-and education-matched groups of participants, these data will thus require further support from wider samples.

In conclusion, the dACC sector of the anterior salience network appears to represent a key node of the functional disorganization underlying slowed decision-making in AUD patients. Distinct frequency bands and spatial sectors of this network indeed displayed a relationship between resting-state metrics and deliberation time, both regardless of group and specifically in AUD patients. By leveraging its neural correlates, these findings provide novel insights into the well-known increase of decision latency in AUD^15,16^, which appears to reflect a decreased temporal synchronicity in networks underlying the engagement of executive processes by behaviourally relevant stimuli such as gambles or, more generally, decisional settings. The core of this impaired neural mechanism seems to involve the dACC sector of the anterior salience network, associated with the “output” motor stage of response selection^35,36^, where heightened activation in AUD—tracking the increase in deliberation time—might represent a compensation mechanism counteracting the concurrent decrease of internal coherent activity.

## Supplementary Information

Supplementary Information 1.

Supplementary Information 2.

Supplementary Information 3.

Supplementary Information 4.

Supplementary Information 5.

## Data Availability

The datasets generated during the current study are available from the corresponding author on reasonable request.
