# Supplementary material for: Increased decision latency in alcohol use disorder reflects altered resting-state synchrony in the anterior salience network
Source: Sci Rep. 2021 Oct 1;11:19581. doi: 10.1038/s41598-021-99211-1 (PMC8486863; doi:10.1038/s41598-021-99211-1)
Supplement: Supplementary file 1 — Supplementary Information 1. [file 41598_2021_99211_MOESM1_ESM.docx]

Supplementary Figure 1. Coherence of intra-network activity: correlation with CGT decision latency in the whole sample.

For the ICs showing a significant correlation with deliberation time, their direction and strength are depicted by the frequency bins reported below the brain sections (p<0.025 corrected). As shown in the scatterplots within color-coded panels, the association between decision latency and coherence of intra-network activity in the whole sample involves either a negative correlation with high coherent activity (low frequency power; blue colour scale) (9,18,30), or a positive correlation with low coherent activity (high frequency power; red colour scale) (64). In the scatterplots, a “plus” (+) sign denotes female participants. Att Ant salience: anterior salience network; Att Dorsal: dorsal attentional network.
