# Supplementary material for: Increased decision latency in alcohol use disorder reflects altered resting-state synchrony in the anterior salience network
Source: Sci Rep. 2021 Oct 1;11:19581. doi: 10.1038/s41598-021-99211-1 (PMC8486863; doi:10.1038/s41598-021-99211-1)
Supplement: Supplementary file 2 — Supplementary Information 2. [file 41598_2021_99211_MOESM2_ESM.docx]

Supplementary Figure 2. Coherence of intra-network activity: latency-by-group interaction.

For the ICs showing a significant latency-by-group interaction, their direction and strength are depicted by the frequency bins reported below the brain sections (p<0.025 corrected). As shown in the scatterplots within color-coded panels, the relationship between decision latency and coherent activity is stronger, in AUD patients vs. controls, in distinct components related to the anterior salience network (16,18). In the scatterplots, a “plus” (+) sign denotes female participants. Att Ant salience: anterior salience network.
